# Finite element analysis of biomechanical effects of percutaneous cement discoplasty in scoliosis

**DOI:** 10.1186/s12891-023-06741-y

**Published:** 2024-04-12

**Authors:** Cunheng Yang, Fumin Wang, Xingxing Huang, Hao Zhang, Shengbo Shi, Fangjun Meng Zhang, Junxiao Gao, Xiaobing Yu

**Affiliations:** Affiliated Zhongshan Hospital of Dalian University, No. 6 Jiefang Street, Zhongshan District, Dalian, Liaoning Province 116001 People’s Republic of China

**Keywords:** Discoplasty, Degenerative disc, Bone cement, Finite element analysis

## Abstract

**Objective:**

To investigate the effect of bone cement on the vertebral body and biomechanical properties in percutaneous cement discoplasty (PCD) for degenerative lumbar disc disease.

**Methods:**

Three-dimensional reconstruction of L2 ~ L3 vertebral bodies was performed in a healthy volunteer, and the corresponding finite element model of the spine was established. Biomechanical analysis was performed on the changes in stress distribution in different groups of models by applying quantitative loads.

**Results:**

Models with percutaneous discoplasty (PCD) showed improved stability under various stress conditions, and intervertebral foraminal heights were superior to models without discoplasty.

**Conclusion:**

Cement discoplasty can improve the stability of the vertebral body to a certain extent and restore a certain height of the intervertebral foramen, which has a good development prospect and potential.

## Introduction

Chronic low back pain (LBP) is a common complaint, more often found in elderly patients, usually secondary to degenerative spine disease [1]. Most patients seek to maintain or keep the functional structure and state of the spine that has developed degenerative diseases, making improving the patient’s quality of life one of the directions surgeons need to explore [2]. It is well-known that transpedicular fixation, interbody fusion cages, and interbody spacers have been used as surgical methods for the treatment of some degenerative lumbar disc diseases [3]. However, there are still many complications and drawbacks to the use of surgery, such as massive bleeding, postoperative infection, pneumonia, cardiopulmonary disease, thrombosis, or urinary tract infection [2, 4, 5].

Varga et al. [6] developed a novel minimally invasive technique called percutaneous cement discoplasty (PCD) using polymethylmethacrylate (PMMA) as an intervertebral spacer to replace the intervertebral spacer plan. Most of the patients who underwent this surgical approach showed good postoperative outcomes, such as reduced back and leg pain and decreased Oswestry Disability Index (ODI) score in the follow-up, Varga concluded that elderly patients with symptomatic dynamic foraminal stenosis and negative disc pressure are suitable candidates for PCD, especially high-risk patients requiring open surgery. Although discoplasty for scoliosis has been clinically proven to have some effect [6–8], there remains a gap at the finite element analysis level of percutaneous cement discoplasty for scoliosis and understanding its mechanical principles and stress distribution helps physicians understand and adjust the optimal treatment strategy. This study aimed to investigate the performance of L2-L3 segments and their discs in degenerative scoliosis and their use of discoplasty under various stress conditions using finite element methods to understand their mechanistic principles and characteristics.

## Materials and methods

### Experimental procedure

Firstly, a healthy volunteer without spinal diseases was selected. Based on the image data provided by the volunteer, the software mimics 21.0 was used to extract the image data of the volunteer and extract the rough model. Then, the rough model was imported into geomagic 2017 software for smoothing, shell extraction, surface fitting and other operations to obtain the processed spinal model. Then enter solidworks 2020 software for assembly, tissue reconstruction, post-processing and other operations, establish a three-dimensional finite element normal model of two-segment spine (L2-L3), and import it into ansys 17.0 software. After the validity and feasibility of the model are verified by the previous in vitro experimental data, adult degenerative lumbar scoliosis was simulated, the height of the right L2 ~ L3 segment was reduced by 30% and the nucleus pulposus volume was reduced by 25% in solidworks 2020 software, which is a primary degenerative scoliosis mentioned in the literature [9], and the disc vacuum phenomenon occurred in degenerative scoliosis was simulated by removing the nucleus pulposus at the L2-L3 disc [7], and then the disc vacuum was set as cement filling to simulate the cement injection for discoplasty, simulating the discoplasty process, measuring the changes in stress, displacement, and intervertebral foraminal height, and analyzing the differences between these two.

### Establishment of model

**Fig. 1:**
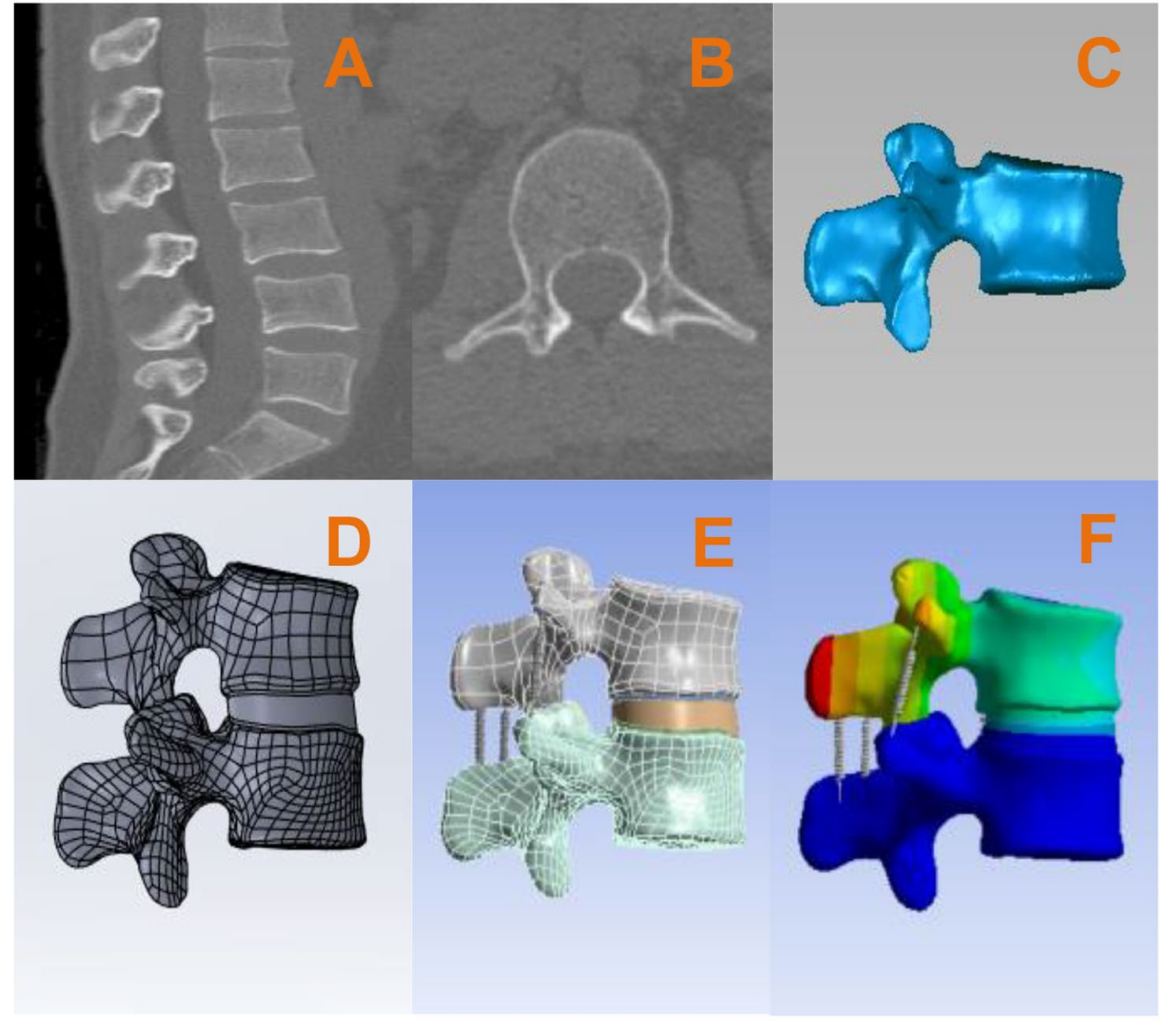
(**A**) (**B**) Original images, (**C**) processing in Geomagic, (**D**) completion of vertebral body assembly and reconstruction of intervertebral discs, (**E**) post-processing such as the reconstruction of ligaments in ANSYS, (**F**) test and evaluation results

We selected a healthy volunteer who had no abnormalities found in imaging data and generated three-dimensional spine geometric models of L2 – L3. The volunteer underwent imaging to obtain information. All CT images were saved in DICOM format. Image data were imported into the medical 3D reconstruction software MIMICS 21.0, three-dimensional models L2 to L3 were extracted, and the vertebral body model was reconstructed from the scanned images and exported to STL format. In order to generate more accurate and smooth 3D digital models, STL-formatted digital geometric models were imported into Geomagic software for smoothing and fitting curved surfaces after the generation and assembly of vertebral bodies, intervertebral discs, and nucleus pulposus using the software SOLIDWORKS. Finally, the model file was imported to ANSYS for mechanical analysis, and the results were obtained. See Fig. 1 for details.

A spring unit was used to simulate the effect of ligaments and joint capsules. The contact relationships between the disc and vertebral bodies, vertebral bodies, ligaments, and between the disc and ligaments are all set as “binding”. The properties of the ligaments were set by stiffness, and the vertebral bodies consisted of 1 mm thick cortical and internal part cancellous bone with 1 mm thick endplates. As for the cortical bone thickness of the vertebral body, some studies [10] performed an examination of the Cortical bone thickness of the lower lumbar spine in specimens, yielding data that the mean shell thickness of the lower lumbar spine was approximately 0.6 mm. Additionally, there are some studies that used a thickness of 1 mm [11] or 1.5 mm [12] in their finite element models, which were derived from CT measurements or some other studies. We set the cortical shell thickness to 1 mm, while the endplate thickness was also set to 1 mm, the articular cartilage was set to 0.3 mm, the disc consisted of nucleus pulposus and outer ring, the nucleus pulposus body product was set to about 40% of the total disc area [13]. The above material properties and assigned values [14–17] are shown in Tables 1 and 2.

**Table 1:** Ligament stiffness value (unit: N/m)

ALL	PLL	LF	CL	ISL	SSL	TL
21	37	25	10	25	34	25

(ALL = anterior longitudinal ligament, PLL = Posterior longitudinal ligament. LF = ligamentum flava, CL = Capsular ligament of joint, ISL = Interspinous ligament, SSL = Supraspinous ligament, TL = transverse ligament)

**Table 2:** The material properties of the finite element model

Component name	modulus of elasticity (MPa)	Poisson’s ratio
Cortical bone	12,000	0.3
*Loosen* cortical bone	8040	0.3
cancellous bone	100	0.3
Loose cancellous bone	33	0.3
Cartilage	10	0.4
Bony endplate	1000	0.4
Normal/degenerated Nucleus pulposus	1.0/8.4	0.499/0.4
Normal/Degenerate Annulus fibrosus	4.2/8.4	0.3/0.4
PMMA	1600	0.33

### Material characteristics and element specifications

Constant loads were used to simulate loading in this study. Under these non-high-strength loads, the simplified bone material changes linearly with a load. With the exception of accidents, most fractures are caused by fatigue and accumulation of injuries. In this study, however, the applied loads were transient, and fatigue attributes could be largely ignored. Therefore, it is sufficient to simulate most of the components with an elastic material model.

### Experimental load

During the movement in the flexion, extension, left and right lateral bending, and left and right axial rotations, an axial force of 300 N was applied downward on the upper surface of the L2 while applying a moment of 10 Nm in this direction and restraining the lower end of the L3 segment.

### Computing facilities

The host of this experiment was configured with GeForce RTX 3080 Vulcan OC graphics card, the CPU was Intel core i9 9900k, and the disk was SAMSUNG MZVLW512HMJP-000H1. Data analysis was performed using SPSS 23.0 software, and statistical differences were considered at P < 0.05.

## Results

### Feasibility validation

Data were obtained and compared with existing literature under six loading conditions (flexion and extension, left and right lateral bending, and left and right axial rotation) [18, 19], and the range of motion of the model in flexion and extension, left and right flexion, and left and right axial rotation was 5.4°, 3.3°, 5.0°, 5.1°, 1.4°, 2.2°, respectively, the minor differences may be due to different bone samples, inconsistent parameter settings, and later processing of the software, which are inevitable, but overall the model data were highly similar to the literature, and good matching between experiment and simulation showed that the finite element model established in this study had high accuracy and verified the effectiveness of this model. This also suggested that our model could be used in subsequent studies.

### Segment Stability

**Fig. 2:**
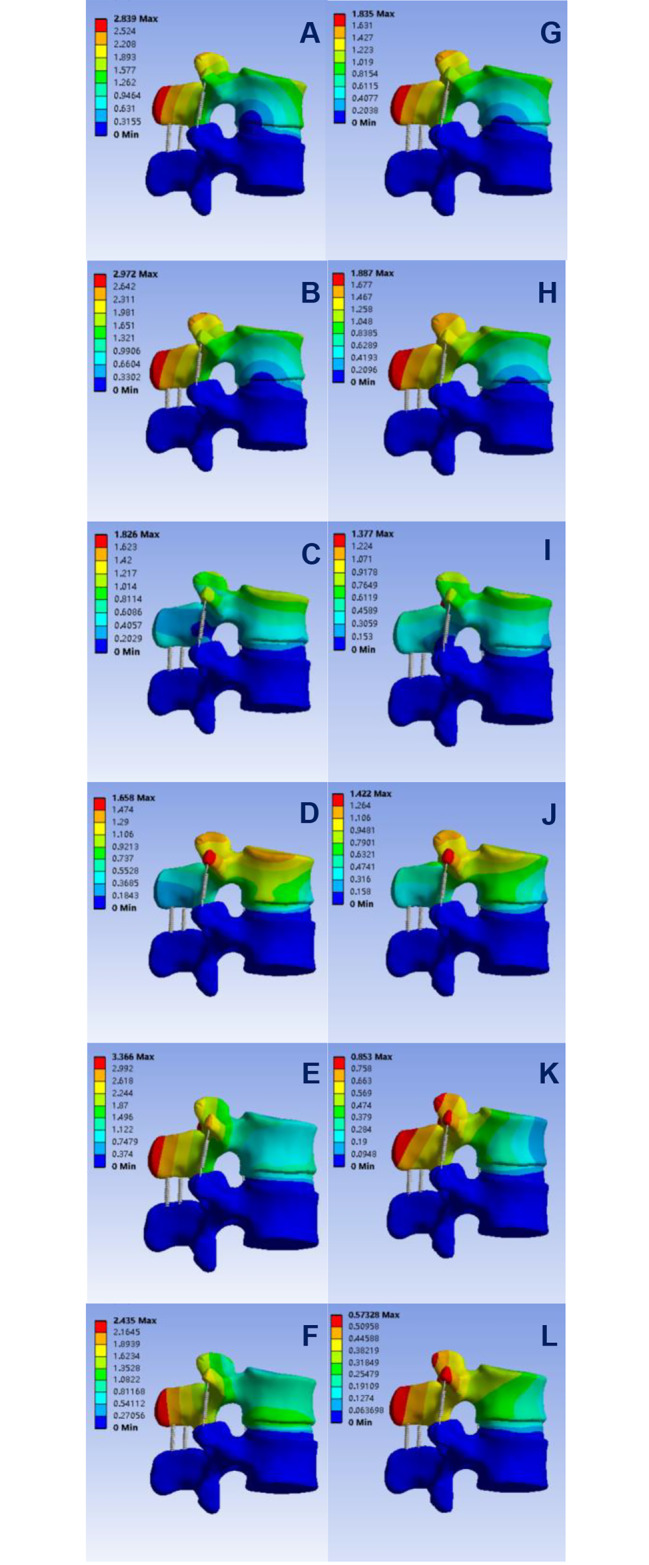
**A, B, C, D, E** and **F** represent the distribution of displacement measured in flexion, extension, left and right lateral bending, and left and right axial rotation before discoplasty, respectively, and **G, H, I, J, K** and **L** represent the distribution of displacement measured in flexion, extension, left and right lateral bending, and left and right axial rotation after discoplasty

Stability is a parameter that needs to be assessed, and vertebral bodies with sufficient stability indicate that the patient is properly treated. After testing the two groups of data of scoliosis vertebrae with vacuum phenomenon and vertebral body after discoplasty, the following data were obtained: the maximum displacement distance of the affected vertebra before surgery in flexion, extension, left lateral bending, right lateral bending, left rotation and right rotation was 2.839 mm, 2.972 mm, 1.826 mm, 1.658 mm, 3.366 mm and 2.435 mm, respectively, and the corresponding maximum displacement distance of the vertebral body after discoplasty was 1.845 mm, 1.887 mm, 1.377 mm, 1.422 mm, 0.853 mm and 0.573 mm, respectively, and the data of the two groups were statistically analyzed and P < 0.05, with statistical differences; and there was also a difference in the range of motion between the two groups. The corresponding range of motion in flexion, extension, left flexion, right flexion, left rotation and right rotation of the affected vertebra was 4.512°, 4.432°, 3.110°, 2.821°, 3.129° and 1.821°, respectively. The corresponding range of motion of the vertebral body was 3.433°, 3.587°, 2.458°, 2.312°, 0.643° and 0.587°. After statistical analysis of the two groups of data, it can be seen that P < 0.05, there is still a statistically significant difference, which confirmed the conclusion that discoplasty could improve spinal stability [6, 7, 20, 21] drawn from the previous literature, indicating that the stability of vertebral model segments after discoplasty is superior. See Fig. 2.

### Extrinsic force

**Fig. 3:**
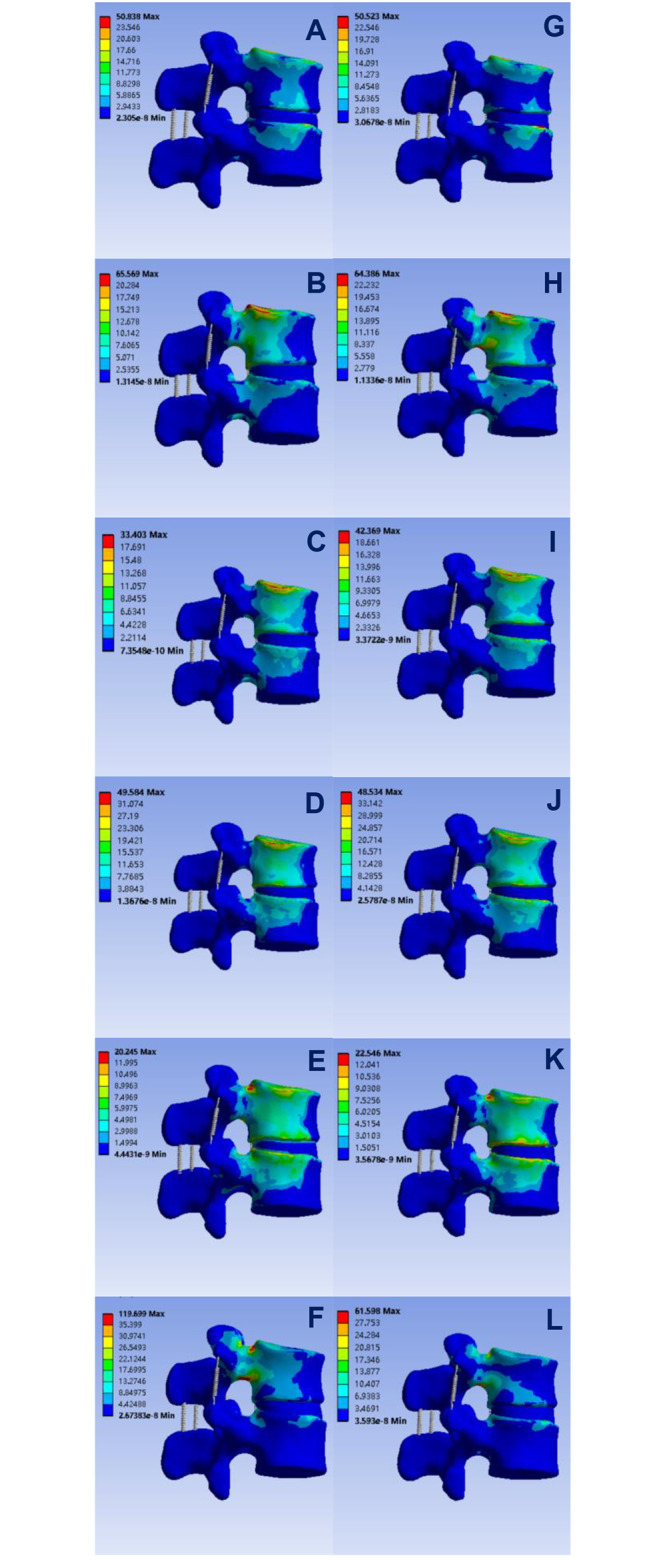
**A, B, C, D, E,** and **F** are the distribution of forces in flexion, extension, left and right lateral bending and left and right axial rotations tests of vertebral segments before discoplasty, respectively, and **G, H, I, J, K, L** are the distribution of forces in flexion, extension, left and right lateral bending and left and right axial rotations test of vertebral segments after discoplasty

The surgery concerns not only the pre-operative vertebral body but also whether this protocol causes sudden changes in the force received and whether it soars above the strength leading to bone destruction. After testing, in terms of flexion, extension, left lateral bending, right lateral bending, left axial rotation and right axial rotation, the maximum stress of the vertebral body model before surgery was 50.838 MPa, 65.569 MPa, 33.403 MPa, 49.584 MPa, 20.245 MPa and 119.699 MPa, respectively, corresponding to the maximum stress of the vertebral body after surgery of 50.523 MPa, 64.386 MPa, 42.369 MPa, 48.534 MPa, 22.546 MPa and 61.598 MPa, see Fig. 3. Thus, the external force received did not change significantly, and even decreased under some conditions.

### Foraminal height

Foraminal height is also important data, and whether it increases or not is of great concern to clinicians [7], which also reflects surgical efficacy to some extent. Varge et al. [6] proposed that discoplasty was a safe and reliable means of maintaining the craniocaudal diameter of the foramen and provided foraminal decompression and correction of lumbar alignment [7]. The foraminal height of the vertebral body model before and after surgery was 19.902 mm, 18.572 mm, 19.497 mm, 18.778 mm, 19.013 mm, 19.336 mm in the former, and 22.300 mm, 20.334 mm, 19.945 mm, 20.076 mm, 20.345 mm, 20.675 mm in the latter, respectively, After statistical analysis of the data between the two groups, it can be seen that P < 0.05, there was a statistically significant difference, indicating that the intervertebral foraminal height of the segments after discoplasty recovered better and could alleviate the loss of intervertebral foraminal height to some extent. See Table 3 After.

**Table 3:** Comparison of foramen heights

	Preoperative vertebroplasty	After vertebroplasty
flexion	19.902 mm	22.300 mm
extension	18.572 mm	20.334 mm
left lateral bending	19.497 mm	19.945 mm
right lateral bending	18.778 mm	20.076 mm
left axial rotation	19.013 mm	20.345 mm
right axial rotation	19.336 mm	20.675 mm

## Discussion

Segmental instability, stenosis, and deformity of the spine as a result of changes in human age are often the cause of adult degenerative scoliosis [22, 23]. The movement of the lumbar vertebra depends typically on the functional structure composed of facet joints and intervertebral discs on both sides to maintain the balance of spine. If this balance is damaged, ligament hypertrophy, osteophyte and facet joint hyperosteogeny will occur [9]. If degenerative scoliosis occurs in the human lumbar spine, it is mostly caused by lumbar degeneration [17]. Degenerative lumbar disc disease is usually caused by asymmetric spinal loading, which in turn leads to the generation of asymmetric degenerative lumbar discs. One of the main causes of low back pain is lumbar disc degeneration in the human spine. Adult degenerative scoliosis is characterized by low back pain, radicular pain, and claudication. The disease is often caused by a variety of causes. The common factors are facet joint degeneration, ligament laxity, disc dehydration, degeneration, and collapse. Often these factors synergistically lead to or induce the occurrence of degenerative spinal diseases. It is of concern that some scholars believe that the health risk of patients will gradually increase with the continuous increase of spinal cobb angle to a certain extent [24, 25], causing many physical and mental health problems, and even causing life-threatening conditions [26].

Currently, the ageing of the population is a serious problem, and people pay more and more attention to medical conditions and quality of life, degenerative scoliosis has become a high concern of health care problems [27]. Surgical intervention is considered to be an effective way to treat degenerative scoliosis, and how to select the best surgical approach is one of the current research hotspots of degenerative scoliosis. Because the planning and modalities of scoliosis disease are variable and complex, there is great uncertainty and diversity in determining the optimal treatment for clinical patients [28–30]. Fusion treatment of the vertebral body is generally considered a feasible option in clinical practice. However, for elderly patients, performing invasive surgical procedures increases the risk of complications such as thromboembolism and deep wound infection, and elderly patients with severe disc diseases are not suitable for prolonged open surgery due to an increased risk of perioperative complications, which is challenging for them to undergo surgery. However, the advent of percutaneous cement discoplasty, as a minimally invasive alternative, It changes the situation that large-scale surgery is often needed, reduces the expected injury of surgery and protects the fragile physical condition of patients, has provided a new option for elderly patients whose physical condition does not allow major trauma but who have the expectation of relief from the pain caused by degenerative scoliosis, So that patients can be effectively treated with only a small wound and do not have to bear the risk of open surgery, which is helpful for patients to recover. Clinicians have put this procedure into the treatment of scoliosis and achieved good results, with varying degrees of improvement in postoperative ODI index and visual analogue scale (VAS) [7, 31]. Discoplasty can also rapidly provide a segmental stabilization effect and indirect decompression due to increased foraminal diameter [6]. It can be seen that percutaneous cement discoplasty has shown certain advantages.

In our current study, finite element models provide a powerful tool to investigate the biomechanics of discoplasty. However, some limitations of finite element methods have to be considered. For simplification, the material representation of the biological structure was assumed. Our finite element model was constructed from normal spinal CT scans, which may differ from the spinal vertebral condition of clinical patients in practice. Therefore, the simulated loads may be different from the actual situation of the patient, which may affect the stress distribution on the spine model. It should also be considered that although there are differences due to the lack of certain parameters and data in vivo or in vitro in some cases, the structural design of the model is too idealized and is different from the actual clinical case, which is inevitable, it can still show the evolutionary trend. In addition, the experimental model is not always the same as the clinical practice, and there will always be differences in individuals. The model restriction conditions also include age, sex, race, actual disease state, and so on, which vary from individual to individual, and more reasonable conditions and loads can also be set to evaluate the situation further.

On the other hand, although PMMA has been put into use by clinicians for a long time, it is generally considered as a safe and non-toxic method, but the use of PMMA will also be accompanied by some risks related to bone cement leakage, and for osteoporosis patients, although the risk and ratio of joint collapse are not too high, it should also attract the attention of clinicians when using this operation, which is indispensable and should be considered by patients. In addition, because PMMA is an inert material, although it is non-toxic, it also lacks the biological activity, osteogenesis induction and tissue regeneration and repair properties of new materials such as titanium implants [32], which is also one of the key points to be studied and improved in the future. We will continue to pay attention to this aspect and deserve our attention.

At the same time, it is worth noting that as for the comparison between percutaneous vertebroplasty and minimally invasive arthrodesis, because the actual adaptation of the two operations is not completely different, and the research literature on percutaneous vertebroplasty is not everywhere, it is still necessary for our researchers to make a separate study and comparison on their adaptation, curative effect, postoperative rehabilitation and biomechanics, which will be a very exploratory topic for us.

Percutaneous cement discoplasty (PCD) provides a more suitable surgical method for some patients with degenerative lumbar scoliosis, greatly reduces the damage caused by traditional surgery, shortens the recovery time required after surgery, reduces the incidence of possible complications caused by postoperative recovery, enhances the stability of vertebral segments, and recovers the height of intervertebral foramen to a certain extent, improves the degree of scoliosis without suddenly increasing the local stress to an unbearable point, this property deserves our attention, this simulation experiment provides some theoretical reference basis for scoliosis discoplasty that has been put into clinical application, indicating that discoplasty has considerable medical potential and broad space in this disease.

## Data Availability

The datasets used and/or analysed during the current study available from the corresponding author on reasonable request.
